# News and Events of the Week

**Published:** 1910-08-20

**Authors:** 


### News and Events of the Week.

Vacation courses for medical students at University College, in chemistry, physics, biology, anatomy, and physiology, begin on September 1.

The following are the arrangements for next week at the West London Postgraduate College : At 10 a.m., August 22 and 25, demonstrations by surgical registrar; at 10 a.m., August 26, demonstration by medical registrar; at 12 noon, August 22, pathological demonstration; at 12.15 p.m., August 23, Dr. Pritchard, clinical pathology; at 12.15 p.m., August 24, Dr. Grainger Stewart, practical medicine. Lectures at 5 p.m. : August 23, Mr. Bidwell, dilation of the sigmoid; August 24, Mr. Addison, hernia in infancy.

"The Odd Sparrow" is the title of this year's annual report of the work of the Ragged School Union and Shaftesbury Society. Those who are interested in child study will do well to procure this interesting and charmingly illustrated booklet, which gives very valuable information on many points which should prove useful to pediatrists. The Society is doing good work and needs all the help it can get. Its offices are 32 John Street, Theobald's Road, W.C.

The Australian State Treasurer (Mr. Watt) (writes a Melbourne correspondent) made an ominous speech recently when discussing the difficulty that almost every charitable institution finds in raising funds. "My experience at the Treasury convinces me that before very long the charity problem will have to be tackled with a view to charity taxation." He would say no more, for probably there rose before his inner vision those grand old pioneers and early statesmen of Australia who were so anxious that none of the evils of the Mother Country should ever be reproduced here that they refused to think of Poor Rate, Workhouse, or Dole. "Our charity must always be voluntary and kind."

The Universities' Mission would like to hear of a medical man who would go to Nyasaland for a period of not less than two years, and work in the diocese under the Bishop. If necessary, the Mission is prepared to give a stipend—in addition to passage (out and home), outfit, board and lodging. The approval of the doctors forming the Medical Board as to medical qualifications, health, etc., would have to be obtained. The Commissaries of the Bishop (or the Bishop himself if still in England) would decide as to the other qualifications. The doctor would be regarded as in every way a member of the staff, and no one would be happy in such a position who was not a communicant member of the Church of England, and in general sympathy with the aims and methods of the Mission. His own actual work, however, would only be the medical care of the staff, the superintendence of the hospitals, and the visiting of patients (Europeans as well as natives). There are seven nurses at Nyasa, and small hospitals or dispensaries at each station; at Likoma (the central station) the out-patients in 1908 numbered 37,268, and 376 cases were treated in the hospital. A certain amount of surgical work is involved. Dr. Robert Howard (of Guy's), who worked in Nyasaland ten years, and has now gone to the Zanzibar diocese, gave certificates to two trained native assistants. Application in the first instance should be made to the Rev. D. Travers, Secretary U.M.C.A., 9 Dartmouth Street, Westminster, S.W.

The inaugural address of the winter session of the London School of Tropical Medicine will be delivered by Professor Miers, of the University of London, under the presidency of Sir West Ridgeway, on Friday, October 14.

Negotiations are now in progress, writes our Melbourne correspondent, between the Commonwealth and State Governments as to what is to be done with the lepers of Australia, who are duly segregated at Brisbane. As quarantine has become a Federal department, the State thinks the Federal health authorities should now become the guardians of the lepers, among whom there is at present one Caucasian.

The sixth "Illustrated Guide to the Museum of the Royal College of Surgeons of England," by Arthur Keith, F.R.C.S., is a useful compilation for visitors and those who do not know the collection. Dr. Keith has paid special attention to the historical and embryological remains. We should much like to know his reasons for supposing that the alleged Napoleonic relics in the museum are authentic. The Guide is very well indexed and adequately illustrated.

The "Studienreise," under the auspices of the Deutsche Zentralkomitee für ärztliche Studienreise, will this year be to Switzerland. The party assembles at Stuttgart on September 1 and visits Ragaz, Flims, Davos, Vulpera, Tarasp, St. Moritz, Zuoz, Pontresina, Sils Maria, Lugano, Montreux, Caux, Glion, Leysin, Evian, and other well-known centres of medical interest, ending at Freiburg i/B. on September 19. All communications with regard to the excursion must be addressed to the central office of the Committee, 134b Potsdamer Strasse, Berlin, W., before the end of August.

An inquest was held at Camberwell on Tuesday last on an upholsterer, who died in the Camberwell Infirmary from cancer. The widow said her husband was sent, by the advice of his doctor, to Guy's Hospital on August 4. She waited a long time without anyone coming to attend to him, and eventually she was told that he would not be seen by a doctor till next morning. Next day he became delirious. On the following Tuesday her husband was placed in the strong room, and the sister asked the witness whether she could not take him away that night. The witness replied that she would have to take him to the infirmary, but she could not take him there that night. The sister then said, "You must come for him as early as you can in the morning, because it is a great expense to the hospital, and we have to have a man for him." Next morning the witness went to the hospital to bring him away, and someone gave her half-a-crown for the cab-fare. She said, "Is he not going away on the ambulance?" and they took no notice of her remark. He died shortly after admittance to the infirmary. The medical officer at Camberwell Infirmary said death was due to heart failure from general exhaustion, following septic poisoning. He did not think that the man ought to have been removed from the hospital, as he was in a dying condition. The jury returned a verdict of "Natural death," without commenting on the circumstances attending the death of the patient.

The Victorian Board of Health has decided to appoint four more medical inspectors at salaries of £250.

Mr. William James Tivy, F.R.C.S., of Victoria Square, Clifton, a Fellow of the Royal Medical Chirurgical Society, of the British Gynæcological Society, London, and of the Obstetrical Society of London, who died on July 12, aged fifty-nine, left estate valued at £2,376 gross.

The Women's Imperial Health Association is commencing a crusade to-day (Saturday), on behalf of the health of the nation, with its first caravan "The Aurora," from which lectures and demonstrations on health, illustrated by biograph pictures and views, will be delivered by competent lecturers in the towns and villages of England. The Association fulfils a national want, and is essentially a women's Association, and it is to the women that its lectures will chiefly appeal. It appeals for no money, for it has at the present ample funds for its immediate objects. No admission fees are charged and no collections made. Pamphlets and leaflets will be freely distributed from the caravan. To-day's ceremony, which will be very picturesque, will consist of a brief exposition of the work and aims of the Association and the display of a few typical biograph films. The caravan will be christened "The Aurora" (signifying "the dawn of a new era") with a flask of pure water. Miss Lena Ashwell, who has kindly undertaken the task, is the wife of Dr. H. J. H. Simson, a member of the Executive Council. Immediately after the ceremony the caravan will proceed up the Thames Valley en route for Bath.

The Watford Coroner held an inquest at the District Hospital on Saturday on Selina Carpenter (44), a patient who died the previous day during an operation under an anæsthetic. Dr. J. D. Burnett, of Watford, said that he had attended the deceased since July 30 for pains in the left side, accompanied by a rise in temperature. On August 10 some unfavourable symptoms developed, and witness called in Dr. C. H. Hall, of Watford, in consultation. They agreed that an operation was the only hope for the patient. Witness explained this to deceased's husband and to the deceased herself, and she consented to go into the hospital for the operation. Witness assisted the surgeon, while Dr. Hall administered the anæsthetic. The operation was opening up the left side over the kidney for the purpose of exposing it and evacuating an abscess. This was done, but just before they commenced stitching up the patient collapsed. They tried to revive her, but were not successful, and she died directly. The Coroner: Did you discuss with Dr. Hall before the operation what anæsthetic should be used? No; ether and gas was what Dr. Hall chose to use. That, of course, was his part. Dr. Hall stated that he examined the deceased in the ward-room previous to the operation. He recognised her desperate condition, but considered that her constitution was able to stand a considerable amount of shock. The operation lasted an hour, and just before it was finished the patient's face suddenly turned quite pale and the pulse became very irregular and weak. Witness at once stopped the operation, and every effort was made to restore animation, without success. The foreman of the jury asked Dr. Hall if the operation was performed satisfactorily and whether everything was done for deceased that could be done. Dr. Hall replied that everything possible was done. Deceased's husband, who was present at the inquiry, said he was perfectly satisfied. A verdict of death from shock consequent upon the operation was returned, and the jury exonerated the doctors from any suspicion of negligence.

### ASSOCIATION OF MEDICAL OFFICERS OF HEALTH.

A general meeting of the Association of Medical Officers of Health was held at the Council Chamber of the British Medical Association offices on July 29, with Dr. F. G. Crookshank in the chair. The Chairman outlined the position that had arisen in consequence of the recent action taken by the representative meeting of the B.M.A., and pointed out that the Council of the Association would probably draft a new resolution relating to part-time Medical Officers of Health, to be submitted to the next annual representative meeting. It was proposed by Dr. Freemantle and seconded by Dr. Wilkinson, that the following resolutions be sent to the Central Council of the B.M.A., and also the Divisions: "This meeting expresses its extreme regret that the representative meeting has not rescinded Minute 234, but welcomes its reference to the Council for reconsideration, and trusts that the whole question will now be reconsidered in the light of the general interests of the medical practitioner and his varied appointments in the public service." It was also resolved: "That in the opinion of this meeting no evidence has yet been adduced to show that the interests of the public health suffer when, as is usually the case, a Medical Officer of Health engages in work other than the restricted duties of his office, and no reason exists for differentiation in respect of conditions of tenure or superannuation between part and whole time medical officers." A vote of thanks was unanimously passed to Drs. Langdon-Down, Freemantle and Taylor, and also to those who had voiced the cause of the part-time Medical Officers of Health at the various meetings of the British Medical Association.

Dr. Parkinson, of Wimborne, suggested the feasibility of a federation of all the various societies of part-time Medical Officers, such as the Poor Law, police surgeons, public vaccinators, &c. He urged that one large society would be much more likely to effect good than many smaller ones. General approval was expressed, and it was agreed to refer the matter to the Council for consideration at their next meeting in October.

In the evening a dinner was held at the Holborn Restaurant, and among those present were Mr. Arnold Ward, M.P.; Dr. Freemantle, County Medical Officer for Herts; Dr. Foulerton, County Medical Officer for Sussex; Dr. Hogarth, County Medical Officer for Buckingham; Mr. George Elliston; Drs. Drury, Gibbs-Smith, Sells, Meredith, Sharman, King, May, Wilkinson, and others. Dr. Crookshank was in the chair. Dr. Foulerton, in proposing the toast of the Association, said that he was opposed to the municipalisation of medicine, which would result if part-time appointments were abolished. In some appointments whole-timers are necessary, but in small districts part-time appointments were better for the public. He had never had any complaints of unfair conduct on the part of part-time men towards the other practitioners of the district. Dr. Crookshank replied, and emphasised the change in professional opinion that had taken place during the year and the importance of the present part-time system to the public as well as to the profession. Mr. Arnold Ward, M.P., also said he was in favour of the general principles of the Association, and individualistic efforts as against socialistic efforts. He advised the Association to obtain the support of their profession in Parliament. Dr. Freemantle proposed the kindred Societies, and Dr. Drury, President of the Public Vaccination Association, replied in sympathetic and cordial terms. The Honorary Secretary (Mr. D. A. Belilios) briefly responded to the toast of the officers.

